# High glucose upregulates connective tissue growth factor expression in human vascular smooth muscle cells

**DOI:** 10.1186/1471-2121-8-1

**Published:** 2007-01-16

**Authors:** Xiaojing Liu, Fengming Luo, Kejian Pan, Wenchao Wu, Huaiqing Chen

**Affiliations:** 1 Institute of Biomedical Engineering, West China Center of Medical Sciences, Sichuan University, Chengdu, China; 2 Laboratory of Cardiovascular Diseases, West China Hospital, Sichuan University, Chengdu, China; 3 Golden-Card Ward, West China Hospital, Sichuan University, Chengdu, China; 4 Department of Biochemistry, Chengdu Medical College, Chengdu, China

## Abstract

**Background:**

Connective tissue growth factor (CTGF) is a potent profibrotic factor, which is implicated in fibroblast proliferation, angiogenesis and extracellular matrix (ECM) synthesis. It is a downstream mediator of some of the effects of transforming growth factor β (TGFβ) and is potentially induced by hyperglycemia in human renal mesangial cells. However, whether high glucose could induce the CTGF expression in vascular smooth muscle cells (VSMCs) remains unknown. Therefore, this study was designed to test whether high glucose could regulate CTGF expression in human VSMC. The effect of modulating CTGF expression on VSMC proliferation and migration was further investigated.

**Results:**

Expression of CTGF mRNA was up-regulated as early as 6 hours in cultured human VSMCs after exposed to high glucose condition, followed by ECM components (collagen type I and fibronectin) accumulation. The upregulation of CTGF mRNA appears to be TGFβ-dependent since anti-TGFβ antibody blocks the effect of high glucose on CTGF gene expression. A small interference RNA (siRNA) targeting CTGF mRNA (CTGF-siRNA) effectively suppressed CTGF up-regulation stimulated by high glucose up to 79% inhibition. As a consequence of decreased expression of CTGF gene, the deposition of ECM proteins in the VSMC was also declined. Moreover, CTGF-siRNA expressing vector partially inhibited the high glucose-induced VSMC proliferation and migration.

**Conclusion:**

Our data suggest that in the development of macrovascular complications in diabetes, CTGF might be an important factor involved in the patho-physiological responses to high glucose in human VSMCs. In addition, the modulatory effects of CTGF-siRNA during this process suggest that specific targeting CTGF by RNA interference could be useful in preventing intimal hyperplasia in diabetic macrovascular complications.

## Background

Diabetes is a major risk factor for the development of cardiovascular disease and could promote cardiovascular diseases via multiple mechanisms [1]. Hyperglycemia, hyperinsulinemia, and dyslipidemia could increase inflammation and proliferation in the atherosclerotic lesions in coronary and cerebral arteries [1,2]. Evidence suggests that high glucose, via various mechanisms, such as increased production of advanced glycation end products, augmented activation of protein kinase C and enhanced generation of reactive oxygen species (ROS), plays a critical role in the development and progression of diabetic cardiovascular complications [2-4]. In addition, elevated glucose concentration is also known to activate a variety of cells to stimulate extracellular matrix (ECM) synthesis [5-7], which is thought to be mediated by inducing transforming growth factor-β (TGFβ)[8,9] and its downstream mediator connective tissue growth factor (CTGF) [10-12]. However, the mechanism why atherosclerosis is accelerated in diabetes is still largely unclear.

Recently, CTGF has emerged as a key factor in vascular remodeling and in the development and progression of atherosclerosis [13,14]. The CTGF gene contains a TGFβ response element in its promoter region and it is thought to be a downstream mediator of the profibrotic effect of TGF-β [10,15]. But CTGF expression is also regulated by cAMP [16], high glucose [11,17], endothelin-1[18] and angiotensin II [18]. High glucose has been known to stimulate CTGF expression in different cell types, including renal mesangial cells and fibroblasts [10,11,17]. However, there are few data about the direct effects of high glucose on ECM protein synthesis and CTGF induction in vascular smooth muscle cells (VSMC). And the connection between high glucose and CTGF expression in VSMC remains unclear. In the view of the increased expression of CTGF in the atherosclerosis, we hypothesized that CTGF might be upregulated by high glucose in VSMCs, and the upregulation of CTGF might contribute to changes of ECM components. In order to test our hypothesis, we examined the influence of high glucose on the CTGF expression in human VSMCs. In previous study, Primary human umbilical vein smooth muscle cells (HUVSMC) have been characterized as a model for investigation of VSMC functions [19]. Therefore, HUVSMCs were used as a model to study the effects of high glucose on the expression of CTGF and other ECM genes by RNA interference and neutralization antibody in this paper. Our data demonstrate that high-glucose-stimulated VSMC growth and migration, as well as the high-glucose-induced ECM components deposition in VSMCs were attenuated by CTGF inhibition, which suggested that therapies targeting CTGF might be useful in preventing intimal hyperplasia in the atherosclerotic lesions in diabetic macrovascular complications.

## Results

### Effect of high glucose on CTGF expression in HUVSMCs

**Figure 1:**
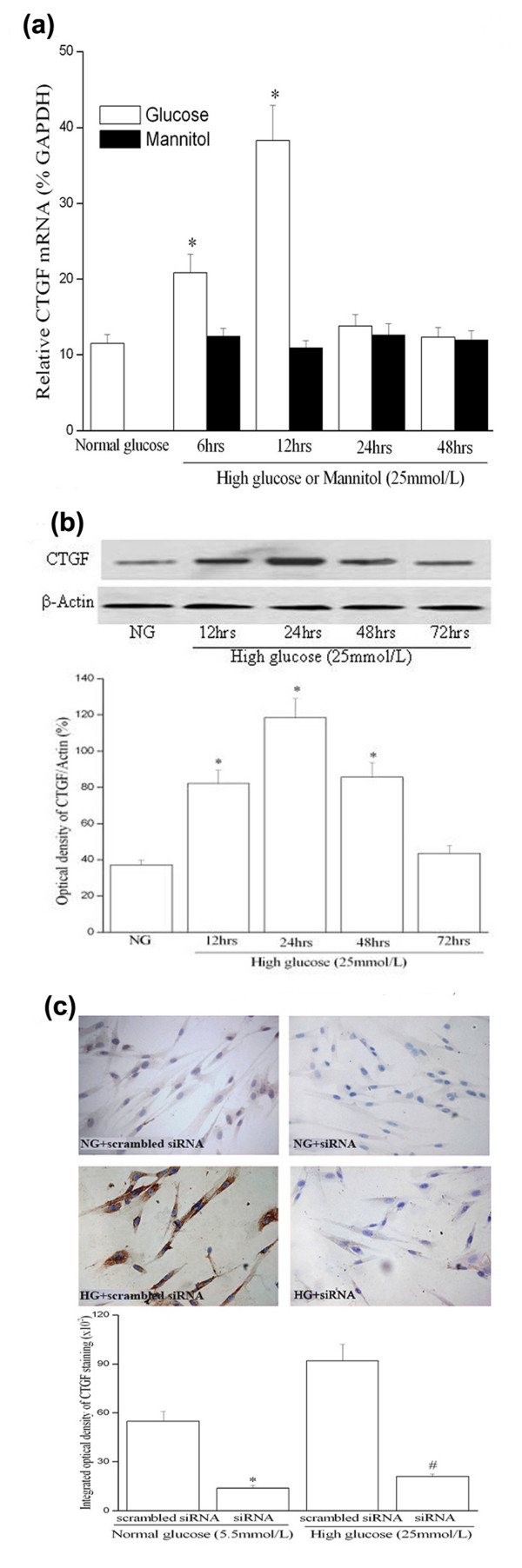
**High glucose increases CTGF mRNA expression (a) and protein production (b, c) in cultured HUVSMCs**. Growth-arrested HUVSMCs were stimulated with high glucose (HG, 25 mmol/L) for different durations. **(a) **Quantitative RT-PCR (Q-PCR) results. Total cellular RNA was isolated from normal glucose (NG, 5.5 mmol/L), high glucose (HG) or mannitol (25 mmol/L) treated HUVSMCs. After reverse transcription, they were subjected to quantitative PCR (Taqman) analysis to determine CTGF mRNA level. Graph is representative of relative CTGF levels in the various conditions. Experiments were performed five times with the similar results (n = 5 in each group). * *P *< 0.05 *vs *NG. **(b) **Representative Western blot (top) and values of total CTGF production (means ± SEM of 3 experiments, bottom). Results of total CTGF protein production were obtained from densitometric analysis and expressed as ratio of CTGF/β-actin. * *P *< 0.05 *vs *NG. **(c) **Immunocytochemical staining of CTGF protein expression in HUVSMCs (top, magnificent of 400×) and integrated optical density (IOD) of the CTGF staining was measured on the images using the Image-Pro Plus software (bottom). Figure shows a representative experiment out of 3 performed experiments. * *P *< 0.05 *vs *scrambled-siRNA transfection under normal glucose media condition. # *P *< 0.05 *vs *scrambled-siRNA transfection under high glucose media condition. *NG: normal glucose; HG: High glucose; scrambled siRNA: scrambled-siRNA plasmid transfection; siRNA: siRNA-CTGF plasmid transfection*.

To determine whether high glucose modulates the expression of CTGF mRNA, HUVSMCs were treated with 25 mmol/L D-glucose, and total RNA was isolated at various times from 6 to 48 hours. Real-time quantitative RT-PCR revealed that high glucose rapidly induced the expression of CTGF above basal levels 6 hours after treatment. The induction of CTGF expression was peaked at 12 hours after treatment, and then declined to near baseline by 24 hours (Figure 1a). To exclude the possibility that high-glucose-induced CTGF expression was caused by increased osmolarity, we tested the effect of 25 mmol/L mannitol on CTGF mRNA expression. Compared with cells in the normal glucose medium, there was no significant stimulatory effect on CTGF expression in HUVSMC cells incubated for 24 hours in normal glucose media containing 25 mmol/L mannitol, confirming the specificity of the high glucose response in stimulating the CTGF expression in HUVSMCs (Figure 1a).

Under serum-starvation condition, growth-arrested HUVSMCs expressed low level of CTGF protein, as shown by Western blot as a band of ≈38 Kda. Total cellular CTGF protein levels began to increase after treated with high glucose for 12 hours, and peaked at 24 hours post-treatment. The elevated CTGF level lasted up to 48 hours after treatment (Figure 1b). The expression of CTGF protein was also analyzed by immunocytochemistry, which showed that growth-arrested HUVSMCs presented a slight CTGF staining, and treatment with high glucose for 24 hours significantly increased cytoplasmic CTGF staining (Figure 1c). These data suggest that high glucose induces both CTGF mRNA and protein production in HUVSMCs.

**Figure 2:**
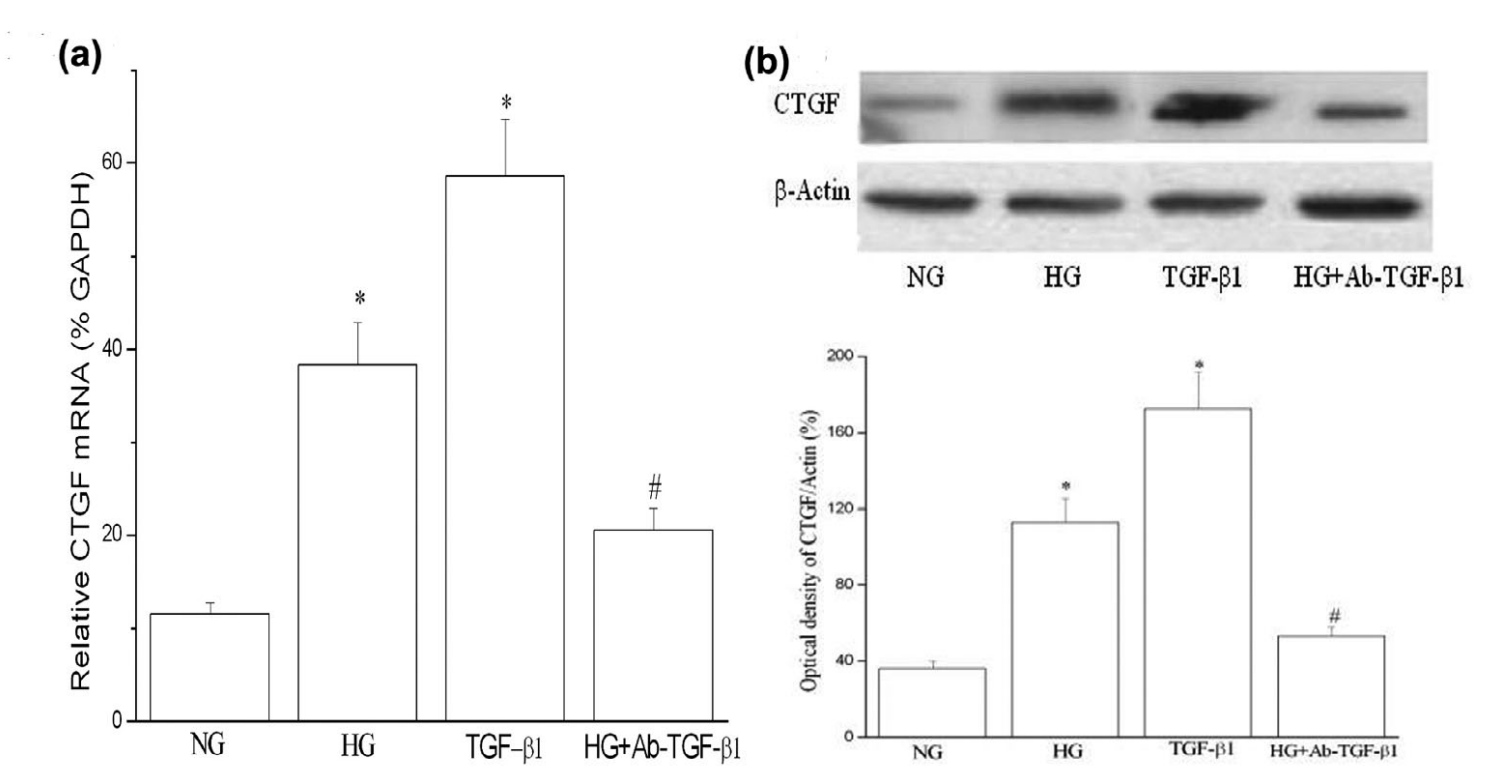
**High glucose-induced CTGF upregulation in HUVSMC is dependent on TGF-β**. HUVSMC cells were co-treated with high glucose (HG, 25 mmol/L) and a TGF-β neutralizing antibody (Ab; 10 μg/mL) for 24 hours. **(a) **Q-PCR results: CTGF mRNA expression was assayed by Q-PCR. Experiments were performed five times with the similar results (n = 5 in each group). * *P *< 0.05 *vs *normal glucose (NG). # *P *< 0.05 *vs *TGF-β 1. **(b) **Representative Western blot of 3 performed experiments (top) and values of total CTGF production (mean ± SEM, bottom). * *P *< 0.05 *vs *NG. # *P *< 0.05 *vs *TGF-β1. *NG: normal glucose; HG: high glucose; TGF*-β*1: TGF*-β*1 treatment (10 ng/mL); Ab-TGF*-β*1: TGF*-β *neutralizing antibody treatment*.

TGF-β has been identified as a potent inducer of CTGF expression and it is also a very important regulator of ECM in different cell types [20,21]. Our results showed that TGF-β treatment (10 ng/mL) also induced CTGF expression in the HUVSMCs. Induction of CTGF by high glucose may occur indirectly, mediated by the action of TGF-β. To test this hypothesis, we examined the effect of a neutralization antibody of TGF-β (10 μg/mL, R&D Systems, USA) on high glucose-induced CTGF expression. We observed that the blockade of TGF-β by a neutralization antibody against active TGF-β partly decreased high glucose-induced CTGF gene and protein production (Figure 2a and 2b). This partial inhibition suggests that endogenous TGF-β synthesis is, at least partly, involved in high glucose-induced CTGF production.

### Role of CTGF in high glucose-induced ECM accumulation in HUVSMCs

**Figure 3:**
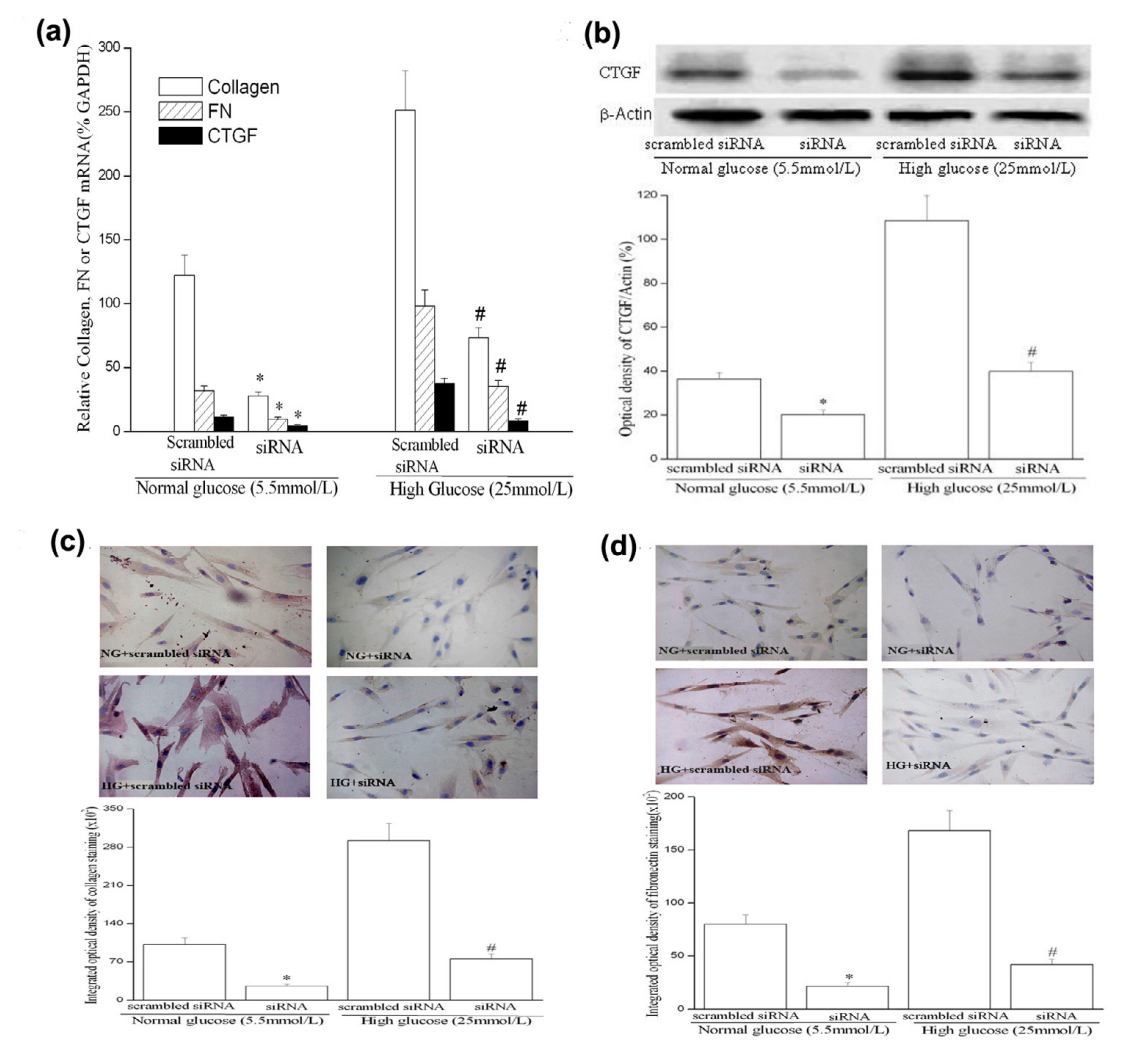
**siRNA-CTGF transfection reduces basal and high glucose-induced CTGF, collagen type I and FN mRNA (a) and protein expression (b, c and d) in HUVSMC. (a) **Q-PCR results: Growth-arrested HUVSMCs were transfected with scrambled or CTGF-siRNA plasmids for 24 hours and then exposed to normal glucose (NG) or high glucose (HG) conditions for 24 to 72 hours. CTGF, collagen type I and FN mRNA expression were assayed by Q-PCR. Experiments were performed five times with the similar results (n = 5 in each group). **(b) **Representative Western blot (top) and values of total CTGF production (means ± SEM of 3 experiments, bottom). Results of total CTGF protein production were obtained from densitometric analysis and expressed as ratio of CTGF/β-actin. **(c) **Immunocytochemistry staining of collagen type I protein expression in HUVSMCs (top, magnificent of 400×) and integrated optical density (IOD) of the collagen type I staining was measured on the images using the Image-Pro Plus^® ^software (bottom). Figure shows a representative experiment of 3 performed. **(d) **Immunocytochemistry staining of fibronectin (FN) protein expression in HUVSMCs (top, magnificent of 400×) and integrated optical density (IOD) of the fibronectin staining was measured on the images using the Image-Pro Plus^® ^software (bottom). Figure shows a representative experiment of 3 performed. * *P *< 0.05 *vs *scrambled siRNA transfection under normal glucose (NG) media condition. # *P *< 0.05 *vs *scrambled siRNA transfection under high glucose (HG) media condition. *Scrambled siRNA: scrambled siRNA plasmid transfection; siRNA: siRNA-CTGF plasmid transfection; NG: normal glucose; HG: High glucose*.

Previous studies have showed that high glucose increased ECM accumulation in cultured smooth muscle cells [9,22]. We therefore investigated whether CTGF was involved in high glucose-induced ECM components deposition, including collagen type I and FN in HUVSMCs. Consistent with other reports [9,22], we observed that ECM components (collagen type I and FN) accumulated significantly under high glucose condition using both real-time PCR and immunocytochemistry analysis (Figure 3).

To block CTGF actions, we used a CTGF-specific small inhibitory RNA construct (CTGF-siRNA) to knockdown CTGF expression. CTGF-siRNA significantly inhibited basal and high glucose-induced CTGF gene expression in HUVSMC as evaluated by quantitative PCR. Similarly, CTGF-siRNA knockdown of CTGF protein was confirmed by Western blot and immunocytochemistry (Figure 3 and 1c). The knockdown of CTGF significantly reduced mRNA and protein levels of collagen type I and FN (Figure 3). As a negative control, the scrambled siRNA had no effect on any of CTGF, collagen type I or FN expression in HUVSMCs (Figure 3a). Thus, the data demonstrate that CTGF is a downstream mediator of high glucose-induced ECM components accumulation in HUVSMCs.

### Role of CTGF in the high glucose-induced proliferation of HUVSMCs

To examine a role of CTGF in high glucose-induced proliferation, we grew quiescent, CTGF gene-silenced HUVSMC cells under high glucose or normal glucose conditions for 48 hours. [^3^H]-thymidine incorporation and cell counting were quantitated in these cells.

**Figure 4:**
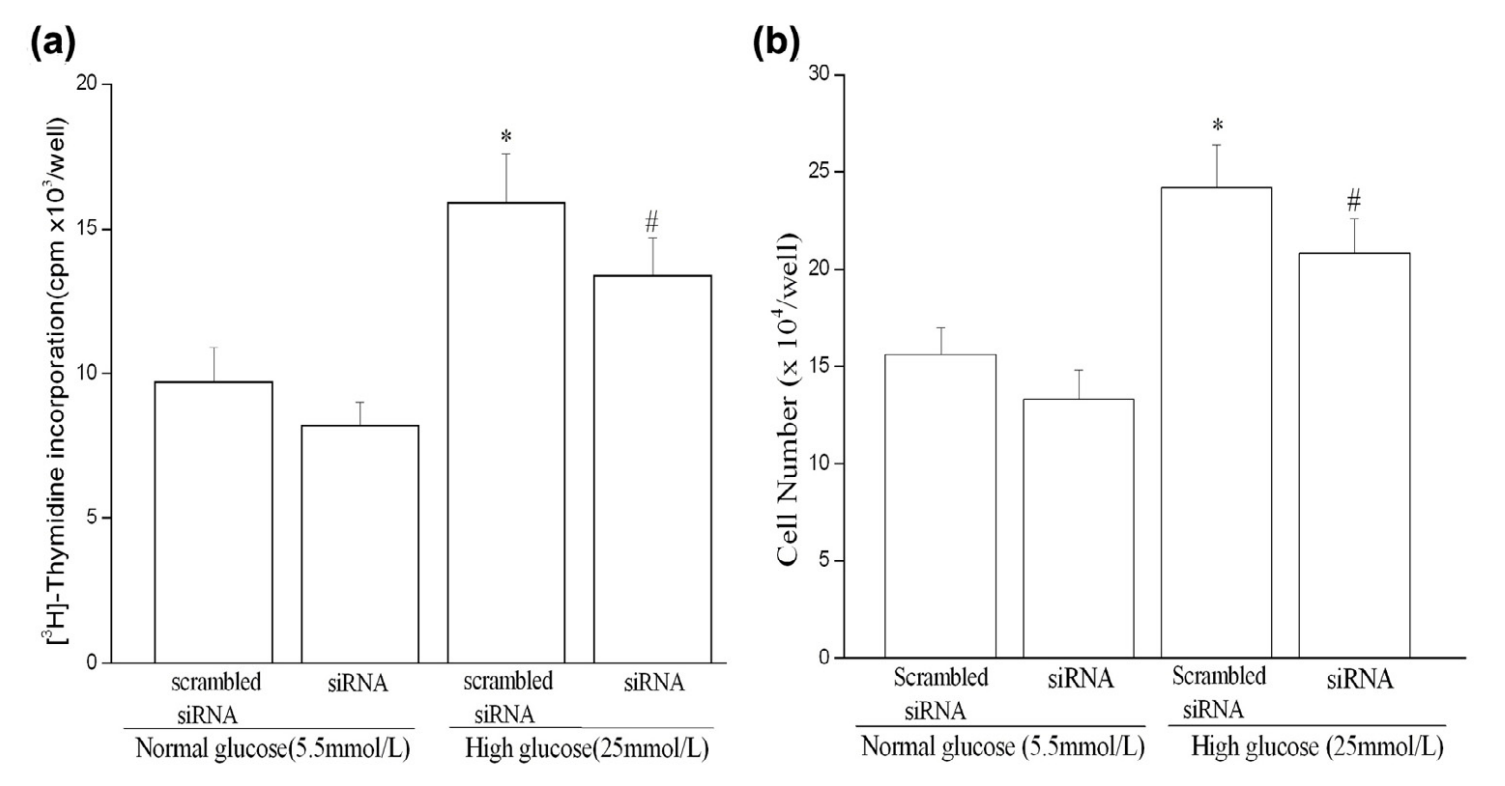
**CTGF is involved in high glucose-induced proliferation of cultured HUVSMCs**. Quiescent cells were transfected with scrambled or CTGF-siRNA expression plasmids for 24 hours and then exposed to HG for 48 hours followed by the assessment of [^3^H]-thymidine incorporation (a) and cell number counting (b). Each value is the mean ± SEM of 6 separate experiments. * *P *< 0.05 *vs *scrambled siRNA transfection under normal glucose (NG) condition. # *P *< 0.05 *vs *scrambled siRNA transfection under high glucose (HG) condition. *Scrambled siRNA: scrambled siRNA plasmid transfection; siRNA: CTGF-siRNA plasmid transfection*.

Figure 4 shows that HUVSMC cells exposed to high glucose conditions was induced a significant 69% increase in [^3^H]-thymidine incorporation compared with normal glucose conditions; and 58% increase in cell number. Our results are consistent with other reports [23,24], which showing that high glucose conditions stimulate the proliferation of cultured VSMCs. To evaluate the contribution of increased medium osmolarity to DNA synthesis, we also examined the effect of 25 mmol/L mannitol on [^3^H]-thymidine incorporation. The [^3^H]-thymidine incorporation in cells incubated 48 hours in normal glucose medium containing 25 mmol/L mannitol was not significantly different from that in the normal glucose medium. This result ruled out the possibility that, the high glucose-induced CTGF up-regulation was caused by increased osmolarity (data not shown). Transfection of CTGF-siRNA in HUVSMC partly prevented the increase in cell proliferation in high glucose (41% inhibition), and to a less extent, in normal glucose medium controls (13% inhibition) (Figure 4). Our data indicate that CTGF is involved in basal and high glucose-induced HUVSMC proliferation

### Role of CTGF in high glucose-induced migration in HUVSMCs

**Figure 5:**
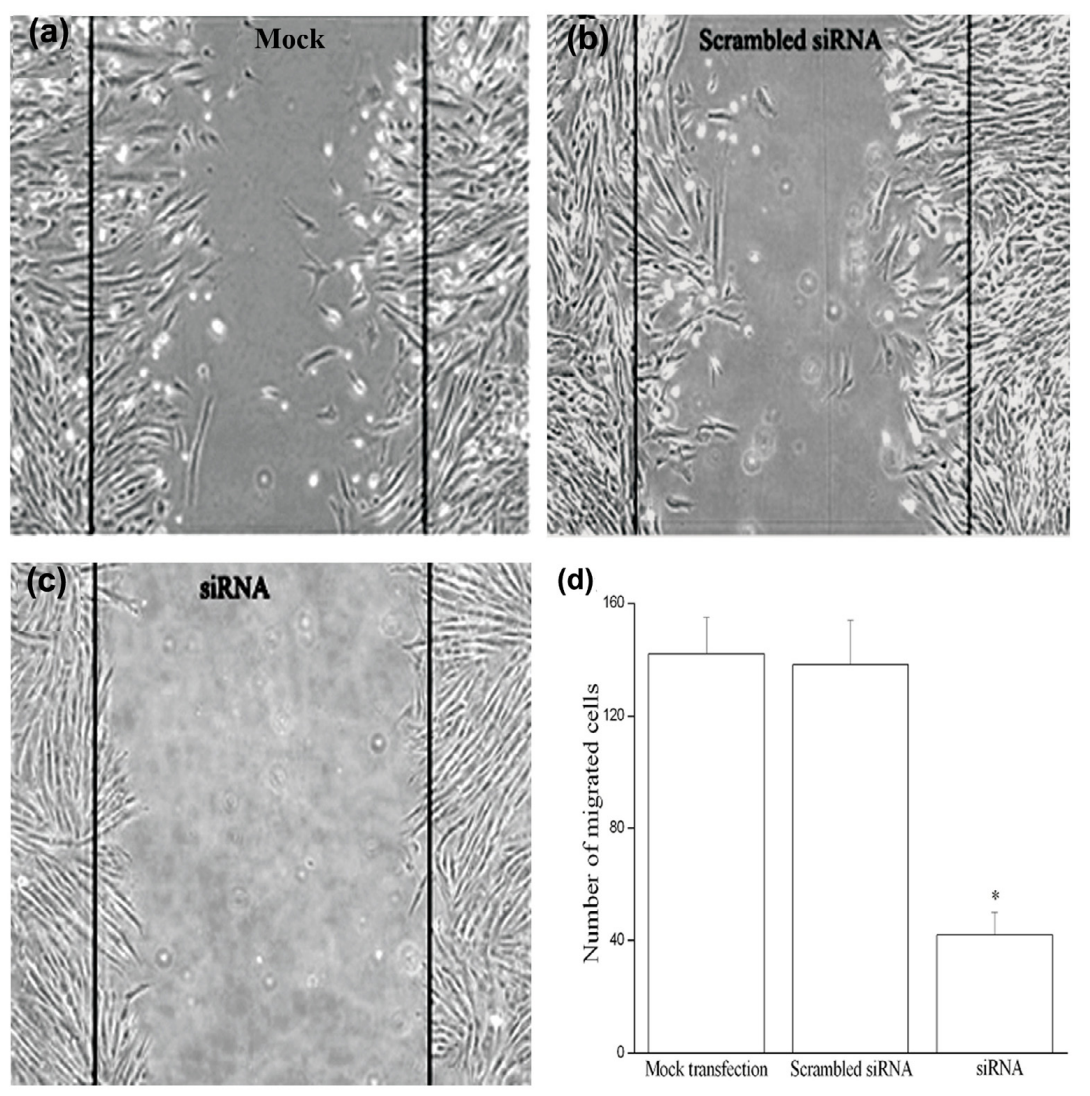
**Role of CTGF in high glucose-induced migration of cultured HUVSMCs**. Quiescent cells were transfected with scrambled or CTGF-siRNA expressing plasmid for 24 hours, then exposed to HG for 48 hours, and followed by the measurement of cell migration in a monolayer scratch wound assay. **Figure (a) **shows a representative result of three mock transfected experiments (Magnification 200×). **Figure (b) **shows a representative result of three scrambled siRNA plasmid transfected experiments (Magnification 200×). **Figure (c) **shows a representative result of three CTGF-siRNA plasmid transfected experiments (Magnification 200×). **Figure (d) **shows the average of migrated cells in three experiments. * *P *< 0.05 *vs *mock transfection or scrambled siRNA transfection.

**Figure 6:**
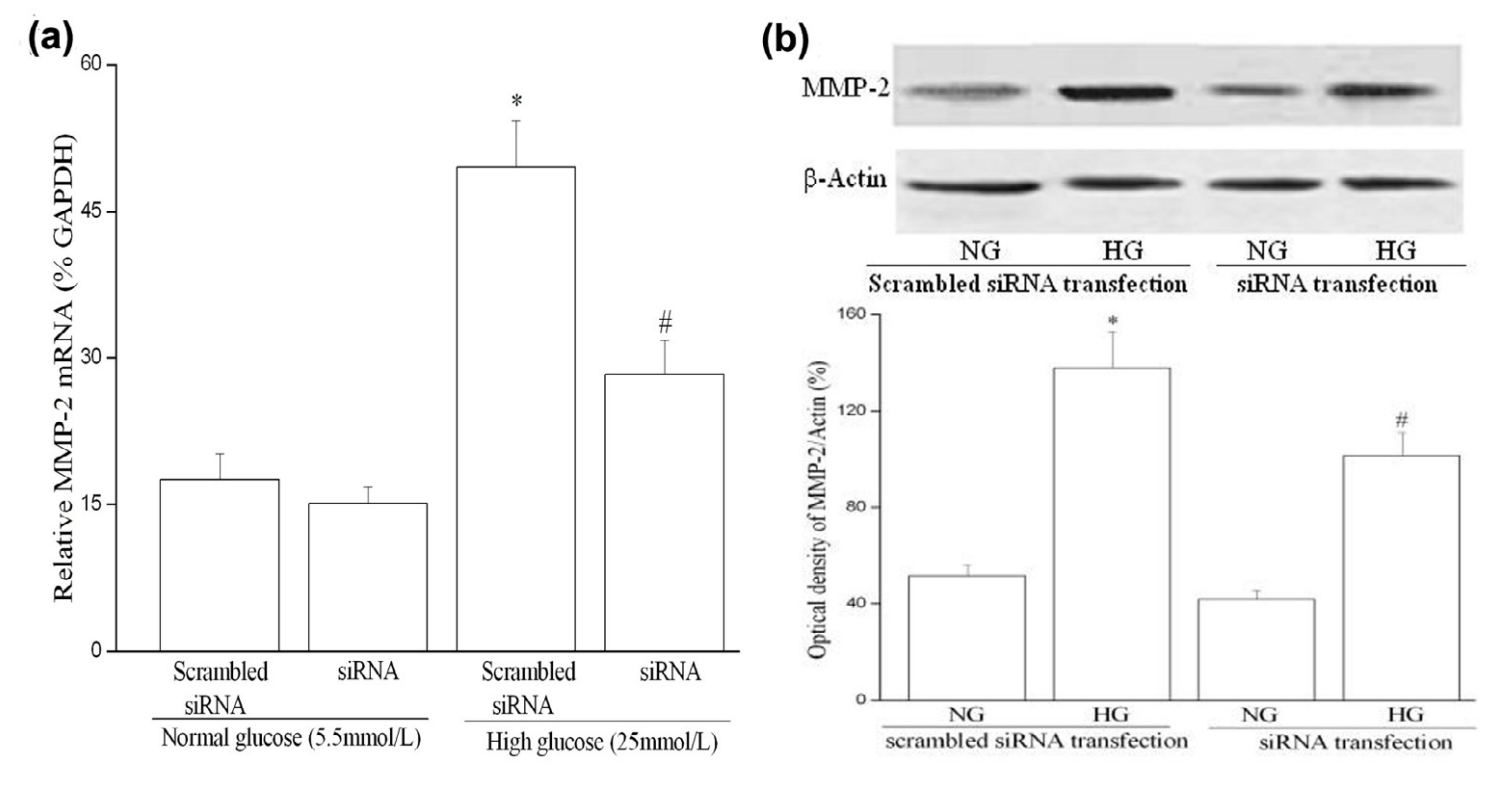
**siRNA-CTGF transfection reduces basal and high glucose-induced MMP-2 mRNA (a) and protein expression (b) in HUVSMC. (a) **Q-PCR (Taqman) results: Growth-arrested HUVSMCs were transfected with siRNA-CTGF plasmid for 24 hours and then exposed to normal or high glucose conditions for 24 hours. 1 μg of total RNA was reverse-transcribed into cDNA and analyzed for expression of MMP-2 mRNA by real-time PCR. Experiments were performed five times with the similar results (n = 5 in each group). **(b) **Representative Western blot (top) and values of total CTGF production (means ± SEM of 3 experiments, bottom). Results of total MMP-2 protein production were obtained from densitometric analysis and expressed as ratio CTGF/β-actin. * *P *< 0.05 *vs *scrambled siRNA transfection under normal glucose (NG) condition. # *P *< 0.05 *vs *scrambled siRNA transfection under high glucose condition (HG). *Scrambled siRNA: scrambled siRNA plasmid transfection; siRNA: CTGF-siRNA plasmid transfection*.

Monolayer scratch wound assays have been used by others to study migration of VSMCs [25,26]. In order to exclusively measure migration, DNA synthesis of HUVSMCs was further blocked by addition of hydroxyurea. Our results showed that 6 hours after injury, the CTGF-siRNA transfected cells were less than the mock transfection or the scrambled-siRNA treated cells to migrate into the wound gap (Figure 5). Furthermore, the expression of matrix metalloproteinase-2 (MMP-2) mRNA and protein were also reduced in the CTGF-siRNA transfected cells. MMP-2 is an important factor directly involved in controlling cell movement and the turnover of ECM [27]. In comparison, the scramble-siRNA transfected cells showed unchanged MMP-2 mRNA expression (Figure 6).

## Discussion

In the present study, the potential correlation between high glucose and CTGF was investigated in cultured HUVSMCs. The major finding of this study is that high glucose up-regulates the expression of CTGF in HUVSMCs and knockdown of CTGF gene results in the inhibition of high glucose-induced VSMC proliferation and migration. These observations establish acritical role of CTGF in mediating high-glucose induced ECM accumulation in VSMC and suggest that inhibition of CTGF may be useful for preventing abnormal VSMC growth and migration in diabetic vessels.

CTGF was first identified as a 38-kDa cysteine-rich protein, which can be specifically induced by TGF-β. It is recently found that CTGF is expressed abundantly in atherosclerotic blood vessels, but only marginally in normal vascular tissues. CTGF is one of the key factors involved in the development of atherosclerotic lesions [13]. To further assess the role of CTGF in diabetic cardiovascular complications, we examined whether CTGF was regulated by high glucose in VSMC. Our data show that exposure of HUVSMC to high glucose, but not iso-osmotic mannitol, leads to an increase of CTGF expression, and the induction of CTGF by high glucose is partly mediated via TGF-β pathway.

Some studies have showed that high glucose may mediate diabetic renal and macrovascular complications by stimulating ECM production [9], and the increased ECM synthesis accounts mainly for intimal plaque formation in the atherosclerotic lesions in diabetic vessels, so the effect of blocking CTGF action on ECM expression was further examined in this study. By CTGF-specific siRNA, our results demonstrate that knockdown of CTGF expression prevents ECM production in VSMC, indicating that CTGF plays an important role in mediating ECM accumulation in VSMC in response to high glucose.

In addition to increased ECM deposition in VSMC, it has been recognized that VSMC proliferation within the vessel wall is another critical pathogenic feature in the development of atherosclerosis. Glucose metabolism has been implicated to play an important role in this cellular mechanism [1]. Neointimal formation, the leading cause of restenosis, is also caused by proliferation of VSMCs. Patients with diabetes mellitus have higher restenosis rates after coronary angioplasty than non-diabetic patients. Enhanced proliferation of VSMC has also been demonstrated in diabetic experimental animal models [24]. In addition, cultured VSMC cells grown in media with high glucose concentration (to mimic hyperglycemia of diabetes) have exhibited increased cell proliferation [23,24] Several intracellular signals elicited by high glucose are responsible for VSMC cell proliferation, including increased expression of TGF-β receptor type II via PKC-β [28], enhanced intracellular ROS production [29], and suppressed apoptosis via upregulation of bcl-xl and bfl-1/A1 levels through PI-3K and ERK1/2 pathways in VSMCs [30]. Our results suggest a role of CTGF in the HUVSMCs proliferation induced by high glucose.

The migration of VSMCs from the media into the neointima is important in the pathogenesis of atherosclerosis. This process is regulated by multiple factors, and it involves changes in the interaction between the ECM and intracellular signaling cascades that regulate cell movement [31,32]. High glucose is one of the multiple factors that could increase VSMCs migration [29,32]. CTGF over-expression can significantly increase the activity of MMP-2 in VSMC conditioned medium and increase the migration of VSMC [25], which suggests a link between high glucose-induced VSMC migration and CTGF over-expression. MMP-2 is able to induce VSMC migration and proliferation in addition to ECM degradation, and it has also been shown to play an important role in atherosclerotic plaque formation and restenosis after vessel injury [33]. Consistent with previous report [34], our data demonstrate that CTGF-siRNA suppresses high glucose-induced HUVSMC migration via, at least partly, down-regulation of MMP-2.

Recently, RNA interference (RNAi) has reinvigorated the therapeutic prospects for inhibiting gene expression and promised many advantages over binding inhibitors, including high specificity. RNAi provides a new, reliable method to investigate gene function and has the potential for gene therapy. In mammalian cells, 21-or 22-nucleotide (nt) RNAs with 2-nt 3' overhangs (small inhibitory RNAs, siRNA) exhibit a RNAi effect [35]. It is important to avoid homologous sequences within a target mRNA in a given protein family [35]. One of the reported CTGF siRNA sequences targets the coding region 360–380 from the first nucleotide of the start codon of CTGF mRNA [36], but it is located within one of the four conserved cysteine rich modular domains-the von Willebrand factor (vWF) domain (307–486 bp) in the CTGF mRNA [37]. In order to construct a specific CTGF-siRNA, we searched for regions of low homology to other genes of the CCN family. With the help of some siRNA-design tools in the Internet, we designed five specific pairs of DNA templates coding siRNA against human CTGF-mRNA and reconstructed the plasmid pSilencer3.1-H1 siRNA-CTGF. However, we only observed one pair of the DNA templates coding the sequence (nucleotides 762–782) has significant effect (79%) to down-regulate the expression of basal and high glucose-induced CTGF expression in HUVSMCs. The reason why only one out of five pairs of siRNA shows specific gene knockdown is unclear. This problem remains to be one of the many challenges of therapeutic usage of siRNA [38]. Down regulation of CTGF markedly reduces the synthesis of high glucose-induced ECM proteins such as collagen type I and fibronectin. Our results indicate that CTGF is involved in ECM accumulation under normal glucose condition, but also it is an important mediator in the ECM deposition induced by high glucose in VSMC. Antagonism of CTGF function could possibly attenuate progression of diabetic macrovascular complications.

## Conclusion

CTGF might be involved in high glucose-induced proliferation, migration and ECM production in VSMC, and could contribute to the pathogenesis of diabetic macrovascular complications. Our results indicate that RNA interference is a useful tool to investigate CTGF gene function and might be useful in developing a potential therapy for diabetic macrovascular diseases.

## Methods

### Cell culture

HUVSMCs and smooth muscle cell medium were purchased from Technoclone (Vienna, Austria). In all experiments, confluent HUVSMC cells at passage 4 to 8 were washed and incubated with serum-free media for 24 hours. These cells were treated with D-glucose at normal glucose (NG, 5.5 mmol/L) or high glucose (HG, 25 mmol/L, as previously used in other reports [17,23]) levels. At the end of their respective incubation periods, cell proliferation, migration and CTGF expression were assessed. Each experiment was repeated at least three times throughout the study.

### Quantitative real-time reverse transcription PCR

The expression of CTGF, collagen type I, fibronectin (FN) and matrix metalloproteinase-2 (MMP-2) gene was identified by quantitative RT-PCR. Total RNA extraction and real-time RT-PCR were performed as previously described [17,39]. Human-specific CTGF, collagen type I and MMP-2 primers and probes were designed using Primer Express Software 1.0(PE Applied Biosystems), synthesized and HPLC purified (Takara, Dalian, China). Primer sequences were as follows: CTGF-F:5'-GCCTGTTCCAAGACCTGT-3'; GCTGF-R: 5'-GGATGCACTTTTTGCCCTTCTTA-3'; CTGF TaqMan probe: 5'-CTCCACCCGGGTTACCAATGAC-3'. Collagen type I (Col1α1)-F: 5'-TGTCGATGGCTGCACGAGT-3'; Collagen type I (Col1α1)-R: 5'-CAACGTCGAAGCCGAATTCCT-3'; Col1α1 TaqMan probe: 5'-CCCCTTGGACGTTGGTGCCC-3'. MMP-2-F: 5'-CCGTGGTGAGATCTTCT-TCT-3': MMP-2-R: 5'-CCTCGTATACCGCATCAATCT-3'; MMP-2 TaqMan: 5'-CACATTCTGGCCTGAGCTCC-3'. GAPDH-F: 5'-GGGTGTGAACCATGAGAACT-3'; GAPDH-R: 5'-CAAAGTTGTCATGGATGACCT-3'; GAPDH TaqMan probe: 5'-CTGCACCAACTGCTTAGC-3'. Human-specific FN primers and probe were synthesized by using the published sequences [40]. For quantification, the target sequence was normalized to the GAPDH mRNA levels.

### Immunocytochemistry

HUVSMC were plated onto coverslips in six-well plates, growth arrested and treated with D-glucose at 5.5 mmol/L or 25 mmol/L levels with or without other compounds. Coverslips were then fixed and blocked as described before [18], followed by exposed to the primary antibodies (anti-CTGF, anti-collagen type I or anti-FN antibody, Santa Cruz Biotechnology, Inc., Santa Cruz, CA, USA). The second antibody was peroxidase-conjugated antibody and the final reaction was visualized with diaminobenzidine (DakoCytomation, Hamburg, Germany), followed by counterstaining with hematoxylin (Sigma-Aldrich). Images were collected using an Eclipse TE2000-U microscope system (Nikon, Japan) and analyzed with Image-Pro Plus^® ^software (Version 4.5, Media Cybernetics, Silver Spring, USA) to semi-quantitatively determine the expression of CTGF, collagen type I or FN.

### Western Blot analysis

Western-blot analysis of CTGF or MMP-2 was performed using rabbit polyclonal antibodies against CTGF or MMP-2 (Santa Cruz Biotechnology, Inc., Santa Cruz, CA, USA), according to the method described before [41,42]. In brief, HUVSMC cell lysates (40 μg) were separated by denaturing 10% SDS-PAGE and then transferred to polyvinylidene difluoride (PVDF) membrane (Millipore) using a MiniProtein III system (Bio-Rad, CA, USA). Transferred proteins were probed with the rabbit polyclonal anti-CTGF or anti-MMP-2 antibodies (1:250) and visualized using the horseradish peroxidase conjugated secondary anti-rabbit (1:3000; Amersham Biosciences) antibody and ECL solution. Equal protein loading was verified by reprobing the membrane with an anti β-actin antibody (Santa Cruz Biotechnology, Inc.). For quantification purposes, densitometric measurements were performed using Quantity One^® ^image analysis software for Windows (Bio-Rad). All specific blot values were corrected forβ-actin expression.

### Plasmid construction and transfection

The pSilencer 3.1-H1 neo siRNA expressing plasmid was purchased from Ambion (Austin, TX, USA). The CTGF-siRNA plasmid expressing short hairpin small interfering RNA (siRNA) under the control of the polymerase-III H1-RNA promoter was produced after inserting pairs of annealed DNA oligonucleotides between the *Bam*HI and *Hind*III restriction sites. The targeted 21-nucleotide (nt) sequences derived from human CTGF mRNA (Genebank: NM_001901; bp 762–781 from the first nucleotide of the start codon) were selected. A scrambled control siRNA with the same nucleotide composition as CTGF siRNA but lacking significant sequence homology to the human genome was also constructed. Transient transfection was performed by use of the cationic lipid Lipofectamine 2000 (Invitrogen, USA) according to the manufacturer's specifications. HUVSMCs were transfected with CTGF-siRNA or scrambled-siRNA expressing plasmids 24 hours before exposure to high glucose concentration.

### Assessment of cell proliferation

[^3^H]-thymidine incorporation and cell counting were used in the assessment of cell proliferation, as described previously [23]. Briefly, HUVSMC cells were subcultured in six-well plates and incubated with serum-free medium for 24 hours. Quiescent cells were transfected with CTGF-siRNA or scrambled siRNA expressing plasmids for 24 hours, then either exposed to normal glucose serum free media or maintained in high glucose serum free media for 48 hours. [^3^H]-thymidine (1 mCi/ml, specific activity 20 Ci/mmol) was added to one set of wells in the last 4 hours of incubation. The other sets of wells were processed for cell counting. For the assessment of [^3^H]-thymidine incorporation, media was removed at the end of incubation, and cells were washed with 10% trichloroacetic acid and digested with 0.5 N NaOH. Radioactivity in the cell digest was counted in a Beckman scintillation counter. [^3^H]-thymidine incorporation is expressed as the total counts per minute per well.

### Scratch wound migration assay

Cell migration was measured using a monolayer scratch injury assay, as described previously [25,26]. Briefly, HUVSMCs were plated at confluence onto glass chamber slides and then transfected with CTGF-siRNA, or scrambled siRNA expressing plasmids for 24 hours. During the last 4 hours, hydroxyurea was added to a final concentration of 5 mmol/L to further prevent DNA synthesis. Following transfection, HUVSMCs were cultured under normal glucose or high glucose serum free media, and a uniform straight scratch was made in the cell monolayer using a 200 μL yellow plastic pipet tip. Monolayers were washed gently, marked (for reference) and photographed using an inverted microscope (Nikon Eclipse TE2000-Y system, Japan). After incubation for 48 hours at 37°C, the cells that have moved into the wound area were quantitated. Four counts were made at various points along each wound that were photographed initially and marked. Migrated cell numbers were derived from the average of these fields in triplicates.

### Statistical analysis

The experimental data were expressed as means ± SEM. Group means were compared by ANOVA using the statistical software program SPSS 10.0 for Windows (Chicago, IL, USA), and *P *value < 0.05 was considered statistically significant in all cases.

## Authors' contributions

XL conceived of the experiments, carried out all experiments and prepared the manuscript. FL conceived of the experiments and performed real-time RT-PCR. KP conceived of the experiments and constructed plasmid vectors. WW performed cell culture. HC provided expert advice and interpretation of the study's results. All authors read and approved the final manuscript.
